# CASP6 predicts poor prognosis in glioma and correlates with tumor immune microenvironment

**DOI:** 10.3389/fonc.2022.818283

**Published:** 2022-09-02

**Authors:** Kai Guo, Jiahui Zhao, Qianxu Jin, Hongshan Yan, Yunpeng Shi, Zongmao Zhao

**Affiliations:** ^1^ Department of Neurosurgery, The Second Hospital of Hebei Medical University, Shijiazhuang, China; ^2^ Department of Neurosurgery, Affiliated Xing Tai People Hospital of Hebei Medical University, Xingtai, China; ^3^ Department of Neurology, Beijing Tiantan Hospital, Capital Medical University, Beijing, China

**Keywords:** CASP6, glioma, pyroptosis, prognosis, immune microenvironment

## Abstract

**Background:**

Glioma is an aggressive tumor of the central nervous system. Caspase-6 (CASP6) plays a crucial role in cell pyroptosis and is a central protein involved in many cellular signaling pathways. However, the association between CASP6 and prognosis of glioma patients remains unclear.

**Methods:**

Four bioinformatic databases were analyzed to identify differentially expressed genes (DEGs) between glioma and healthy tissues. Eighty-one protein-coding pyroptosis-related genes (PRGs) were obtained from the GeneCards database. The pyroptosis-related DEGs (PRDEGs) were extracted from each dataset, and *CASP6* was found to be aberrantly expressed in glioma. We then investigated the biological functions of CASP6 and the relationship between *CASP6* expression and the tumor microenvironment and immunocyte infiltration. The half maximal inhibitory concentration of temozolomide and the response to immune checkpoint blockade in the high- and low-*CASP6* expression groups were estimated using relevant bioinformatic algorithms. Quantitative real-time reverse transcription PCR and western blotting were carried out to confirm the different expression levels of CASP6 between human astrocytes and glioma cell lines (U251 and T98G). We determined the role of CASP6 in the tumorigenesis of glioma by knocking down *CASP6* in U251 and T98G cell lines.

**Results:**

We found that *CASP6* was overexpressed in glioma samples and in glioma cell lines. *CASP6* expression in patients with glioma correlated negatively with overall survival. In addition, *CASP6* expression correlated positively with the degree of glioma progression. Functional analysis indicated that CASP6 was primarily involved in the immune response and antigen processing and presentation. Patients with high *CASP6* levels responded more favorably to temozolomide, while patients with low expression of *CASP6* had a better response to immunotherapy. Finally, *in vitro* experiments showed that *CASP6* knockdown inhibited glioma proliferation.

**Conclusions:**

The pyroptosis-related gene *CASP6* might represent a sensitive prognostic marker for patients with glioma and might predict their response of immunotherapy and temozolomide therapy. Our results might lead to more precise immunotherapeutic strategies for patients with glioma.

## Introduction

Glioma, which is derived from the neuroepithelial cell layer, is the most common cancer of the central nervous system (CNS). In 2016, the World Health Organization classified glioma into four histopathological grades on the basis of the degree of its progression. Grades I and II are defined as low-grade glioma (LGG), while grades III and IV are defined as high-grade glioma. Oligodendrogliomas and astrocytomas belong to the grade II class. Anaplastic oligodendrogliomas, anaplastic astrocytomas, anaplastic oligoastrocytomas, and anaplastic ependymomas are classified into grade III. Glioblastoma (GBM) is grade IV, which is the most malignant type of glioma (1). Surgical resection plus radiotherapy and chemotherapy are the mainstay therapeutic strategies to treat glioma patients. Due to the high aggressiveness, high recurrence rate, and resistance to radiotherapy and chemotherapy the overall survival (OS) of glioma patients is low (2), Despite the progress for glioma research in recent years no major breakthroughs have been made to improve glioma prognosis (3).

Pyroptosis is a form of programmed cell death characterized by cell swelling, lysis, and the release of pro-inflammatory factors (4). Recently, research has shown that pyroptosis plays a crucial role in inhibiting tumor cell proliferation and tumor growth in many kinds of cancer, such as colon cancer (5), non-small cell lung cancer (6), and hepatocellular carcinoma (7). In the field of glioma research, many potential treatments might exert an antitumor effect *via* pyroptosis. For example, the natural nutrient kaempferol was found to be an anti-glioma drug that possibly induces pyroptosis (8). MicroRNA miR-214 and circular RNA hsa_circ_0001836 were found to inhibit glioma growth *via* inducing pyroptosis (9, 10). Caspase 6 (CASP6) is activated in pyroptosis cascade and plays a critical role in this process. Caspase-6 can induce the activation of NLRP3 inflammasome, which is a core step of pyroptosis (11). Accumulating experimental evidence suggests that the apoptosis of hTERT-positive malignant glioma cells is markedly promoted by the induction of the hTERT/rev-caspase-6 complex (12). These findings indicated that CASP6 might play a critical role in the occurrence of glioma and could be a potential therapeutic target. However, the effects of CASP6 on glioma pyroptosis and its mechanism need further investigation.

In this study, we sought to identify pyroptosis-related differentially expressed genes (PRDEGs) through analyzing sequencing datasets obtained from glioma patient tissues. These analyses identified *CASP6* as one of the glioma-associated PRDEGs. Furthermore, we found that *CASP6* expression level was correlated with prognosis of glioma patients, and outcomes of chemotherapy and immunotherapy. In addition, we analyzed the biological functions of CASP6 in glioma and found that CASP6 was involved in the immune microenvironment and the infiltration of immune cells. Finally, the abnormal expression of *CASP6* in gliomas was verified using an external database and cell experiments. Our findings suggest that CASP6 is a marker to predict the prognosis of glioma patients and might be a potential target to treat glioma.

## Materials and methods

### Data collection and preparation

A total of 180 (23 non-tumor and 157 tumor) mRNA expression profiles from patients with glioma were collected from the Gene Expression Omnibus (GEO) database (GSE4290) (https://www.ncbi.nlm.nih.gov/). Data for 1018 patients with glioma were downloaded from the Chinese Glioma Genome Atlas (CGGA) database (http://www.cgga.org.cn/index.jsp). Non-tumor (n = 28) and tumor (n = 522) samples from patients with glioma in the GEO database (GSE108474), termed The Repository of Molecular Brain Neoplasia Data (REMBRANDT), were included. Biological information from patients with glioma (523 with LGG and 171 with GBM) and information from normal brain tissue were obtained from the UCSC Xena project (https://xena.ucsc.edu/). All raw data from The Cancer Genome Atlas (TCGA) (https://portal.gdc.cancer.gov/) and the Genotype-Tissue Expression (GTEx) database (https://www.gtexportal.org/home/) were recalculated using standard pipeline algorithm from the UCSC Xena project. This process minimized the discrepancy between expression data and made the digital data more compatible. Recurrent samples, secondary samples, non-glioma samples, and samples with incomplete clinical information were excluded. A total of 1920 primary glioma samples (TCGA: 662; GSE4290: 153; REMBRANDT: 454 CGGA: 651) and 257 normal tissues (TCGA-GTEx: 206; GSE4290:23; REMBRANDT:28) were included in this study.

### Identification of differentially expressed genes related to pyroptosis in three databases

TCGA-GTEx, GSE4290, and REMBRANT datasets were separately analyzed to detect differentially expressed genes (DEGs) (**Supplementary Figure 1**). These analyses were performed using the R software version 4.1.0 (13). We set |log fold change (FC)|>1 and an adjusted P-values (p-adj) < 0.05 as the thresholds. The DEGs of TCGA-GTEx, GSE4290 and REMBRANT database were confirmed. We retrieved 81 protein-coding PRGs (Relevance Score>1) from the GeneCards database (https://www.genecards.org/) (**Supplementary Table 1**).

### Validation of the identified biomarker

We estimated the prognostic value of *CASP6* in patients with glioma using the CGGA dataset as the validation dataset. Based on the median expression level, the cleaned data were divided into two groups: the high *CASP6* expression group and the low *CASP6* expression group. Survival analysis between the two groups was implemented in the R software using the “survival” and “survminer” packages. Finally, we built receiver operating characteristic (ROC) curves to evaluate the predictive efficacy of *CASP6*.

### Functional enrichment analyses of *CASP6*

Hallmark, Gene Ontology (GO) enrichment analysis, and Kyoto Encyclopedia of Genes and Genomes (KEGG) pathway analysis were performed using the R package “clusterProfiler” (14). Gene set enrichment analysis (GSEA) was used to explore the potential regulatory mechanisms of *CASP6*. We selected the annotated gene sets “h.all.v7.4.symbols.gmt” obtained from the Molecular Signatures Database (MSigDB3) (15), as the reference gene sets. Visualization of the above results was carried out using the R package “enrichplot”. We set 0.05 as the cutoff point for the adjusted *p*-value.

### Prediction of the chemotherapy and immunotherapy response

The response to temozolomide chemotherapy of each patient with glioma in the CGGA was estimated using the “oncoPredict” R package (16). In this analysis, the Genomics of Drug Sensitivity in Cancer 2 (GDSC2) database (https://www.cancerrxgene.org/) was used as the training data. Meanwhile, this algorithm calculated the half maximal inhibitory concentration (IC50) of temozolomide. The Tumor Immune Dysfunction and Exclusion (TIDE) algorithm using a python (version 3.8.6) script (17) was used to evaluate the response to immune checkpoint blockade (ICB) agents.

### Correlation analysis of immune infiltration and CASP6

The “Estimation of STromal and Immune cells in Malignant Tumors using Expression data” (ESTIMATE) (18) algorithm was adopted to predict the level of immune cell infiltration across different *CASP6* expression groups in glioma. The immune score in *CASP6* high- and low-expression groups was determined based on the ESTIMATE analysis. Furthermore, to explore the influence of *CASP6* on the TIME in glioma, we exploited the CIBERSORT (19), single sample gene set enrichment analysis (ssGSEA) (20) and Tumor Immune Estimation Resource (TIMER) (21) algorithms to calculate the infiltration fractions of 22 types of tumor-infiltrating immune cells.

### Verification of CASP6 expression in the Human Protein Atlas

CASP6 immunohistochemical images of normal brains and glioma tissues were downloaded from the Human Protein Atlas (HPA) (http://www.proteinatlas.org). We provide the links to these images in **Supplementary Table 2**.

### Cell culture

Human astrocytes (HAs) were cultured with HA culture medium (Astrocyte Medium) (both from ScienCell Research Laboratories, Inc. (San Diego, CA, USA)). And the glioma cell lines (U251 and T98G) were obtained from Procell Life Science & Technology Co., Ltd. (Wuhan, China). Roswell Park Memorial Institute (RPMI) -1640 medium (Gibco, Thermo Fisher Scientific, Shanghai, China) was used as the basal culture medium of U251 cells, while the basal culture medium of T98G was minimal essential medium (MEM) (Gibco, Thermo Fisher Scientific, Shanghai, China). U251 cells were cultured with RPMI-1640 medium supplemented with 10% fetal bovine serum (FBS) and 1% penicillin–streptomycin (P/S) (Biological Industries at Sartorius, Kibbutz Beit-Haemek, Israel). T98G cells were maintained in the presence of MEM, 10% FBS and 1% P/S. HAs, U251, and T98G cells were cultured in a sterile cell incubator at 37°C with 5% CO2.

### Quantitative reverse transcription real-time PCR

Total RNA from HA, U251, and T98G cells were extracted using Superbrilliant™ 6 min High-quality RNA Extraction Kit (Zhongshi Gene Technology, Tianjin, China, Cat. No.: ZS-M11005). cDNA synthesis was carried out using the Supersmart ™ 6 min 1st Strand cDNA Synthesizer Kit (Zhongshi Gene Technology, Cat. No.: ZS-M14003). QPCR was performed with Supersmart 5xFast SYBR Green qPCR Mix Kit (Zhongshi Gene Technology, Cat. No.: ZS-M13001) on Bio-Rad Laboratories CFX Connect (TM) Real-time PCR Detection System (Bio-Rad, Hercules, CA, USA). The primers were obtained from Thermo Scientific (Shanghai, China), and included those amplifying *CASP6* (forward 5′-AGGTGGATGCAGCCTCCGTTTA-3′, reverse 5′-ATGAGCCGTTCACAGTTTCCCG-3′); *GAPDH* (encoding glyceraldehyde-3-phosphate dehydrogenase) (Forward: 5′-GCAGGGGGGAGCCAAAAGGG-3′, reverse: 5′-TGCCAGCCCCAGCGTCAAAG-3′). Relative mRNA levels were calculated using the 2^-△△Ct^ method (22). Each experiment was carried out independently three times.

### Western blotting

Cells were transfected with the indicated plasmids for 4 h. 72h after the transfection, cells were collected, and lysed using Radioimmunoprecipitation assay (RIPA) buffer and protease and phosphatase inhibitors (Solarbio Science and Technology, Beijing, China). Proteins were separated using 10% sodium dodecyl sulfate polyacrylamide gel electrophoresis (SDS-PAGE;Solarbio Science and Technology), then transferred to polyvinylidene fluoride membranes (Millipore, Billerica, MA, USA). The membranes were blocked using 5% skim milk for 2 h and then incubated at 4°C for 12 h with the primary antibodies recognizing the following proteins: CASP6 (ABclonal Technology, Wuhan, China, Catalog NO: A19552) and α -Tubulin (Abways Biotechnology, Shanghai, China, Catalog NO: AB0049). Secondary goat anti-rabbit IgG antibodies (Abways Biotechnology, Catalog NO: AB0101) were then incubated with the membrane for 1 h at 25°C. The ECL Western Blotting Substrate (Solarbio Science and Technology, Catalog NO: PE0010) was used to visualize the immunoreactive proteins, which were detected and analyzed using the BioRad ChemiDoc imaging system (Bio-Rad, USA).

### Cell Counting Kit-8 assay

U251 and T98G glioma cells were cultured in T25 cell culture flasks. When the cell density reached about 60%, the culture medium was replaced by serum-free medium. *CASP6* small interfering RNAs (siRNAs) were purchased from Zhongshi Gene Technology. The sequence of si-CASP6#1 (Lot:2146812) was 5’-GACUUCCUCAUGUGUUACUCUdTdT-3’ and 5’-AGAGUAACACAUGAGGAAGUCdTdT-3’. The sequence of si-CASP6#2 (Lot:2146814) was 5′-CCUUUGGAUGUAGUAGAUAAUdTdT -3’ and 5’-AUUAUCUACUACAUCCAAAGGdTdT -3’. The sequence of si-CASP6#3 (Lot:2146816) was 5’-GCUUUGUGUGUGUCUUCCUGAdTdT -3’ and 5’-UCAGGAAGACACACACAAAGCdTdT -3’. The CASP6 siRNAs and prepared GP-transfect-Mate reagent (GenePharma, Shanghai, China, Cat. No.: G04009) were added to the T25 cell culture flasks. Six hours after transfection, the medium was replaced by complete medium. After 48 h of incubation, the cells were collected and seeded in 96-well plates (5000 cells/well). After the cells were incubated for 2 h, 10 μL CCK-8 reagent (Report bio&technology Co. Ltd, Shijiazhuang, China, Cat.No.:RP-RC3028) was added into each well and incubated in a cell culture incubator for 1 h. A microplate reader (Synergy H1, Biotek, USA) was then employed to measure the absorbance of the medium in the well. This result was recorded as the results of day 0. The assay was repeated at 1, 2, and 3 days after seeding in 96-well plates. Six replicates were set for each sample. And the experiments were repeated independently three times.

### Colony formation assay

48 hours after transfection, the *CASP6* siRNAs transfected glioma cells (U251 and T98G) in good growth status were collected and seeded in 35-mm dishes at 500 cells/dish. The cells were cultured with complete medium in a sterile cell incubator at 37°C with 5% CO2 for 14 days. The medium was replaced every 2 days. After 14 days of incubation, the colonies were formed. The culture medium was aspirated off, and 800 μL of 4% paraformaldehyde was added in the dish for 40 minutes to fix the cells. Finally, colonies were stained using crystal violet for 20 minutes and counted under a microscope. The experiments were repeated independently three times.

### Statistical analysis

Data analysis and statistical tests were performed using R (version 4.1.0). Comparisons between two groups were performed *via* a Wilcoxon rank-sum test. We conducted statistical analysis of categorical variables between groups using the chi-squared test. The OS analysis of patients with glioma was carried out using the Kaplan-Meier method. The independent prognostic value of *CASP6* and other clinical characteristics were calculated separately using univariate and multivariate Cox regression analyses. Correlation analysis was conducted using the Pearson correlation test. We used the R package “meta.” to determine a pooled hazard ratio (HR) by invoking the random-effects meta-analysis model. A *P-*value < 0.05 was considered statistically significant.

## Results

### Analysis of pyroptosis-related differentially expressed genes in glioma patients

**Figure 1:**
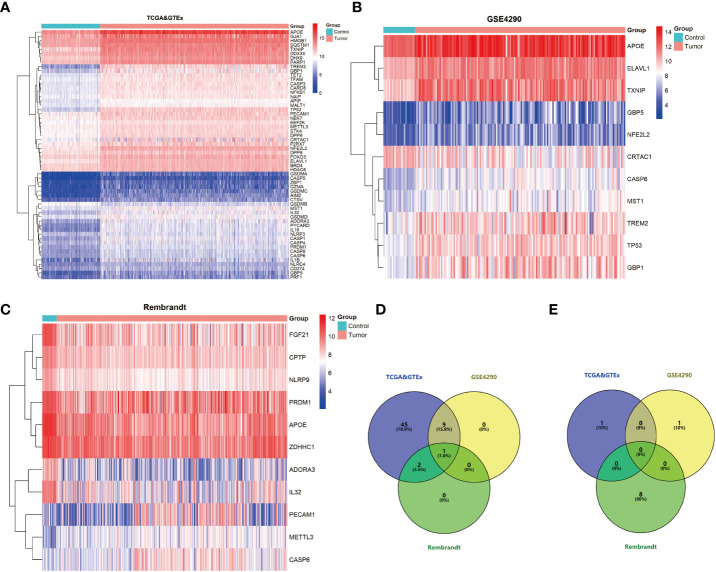
Pyroptosis-related differential expressed genes (PRDEGs) in glioma. **(A)** Heatmap of PRDEGs in the TCGA & GTEx datasets. **(B)** Heatmap of PRDEGs in the GSE4290 dataset. **(C)** Heatmap of PRDEGs in the REMBRANDT database. **(D)** Venn diagrams of up-regulated PRDEGs. **(E)** Venn diagrams of down-regulated PRDEGs.

To identify PRDEGs in gliomas, a collection of 81 genes related to pyroptosis were collated from GeneCards database. Their expression was examined in datasets from GSE4290 REMBRANDT, and the TCGA-GTEx cohorts. In the GSE4290 dataset, 11 PRDEGs were identified, among which 10 were upregulated and one was downregulated. In the REMBRANDT dataset, 11 PRDEGs were uncovered, including three that were upregulated and eight that were downregulated. In TCGA-GTEx dataset, 57 PRDEGS were upregulated and one PRDEG was downregulated (**Figures 1A–C**). Overlap of PRDEGs from these cohorts demonstrated that CASP6 was the only one upregulated in all three datasets, while there was no common downregulated PRDEG (**Figures 1D, E**). Together, these results identified CASP6 as a candidate biomarker in glioma.

### *CASP6* expression could predict prognosis in the CGGA dataset

**Figure 2:**
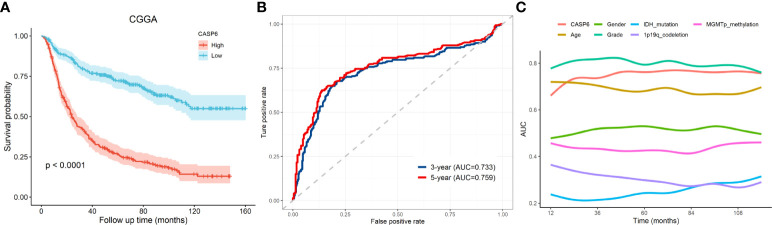
The prognostic value of *CASP6* in the CGGA database. **(A)** Kaplan-Meier survival analyses of overall survival and *CASP6* in patients with glioma in the CGGA database. **(B)** ROC curve analysis to evaluate the prognostic value of *CASP6* expression in glioma in terms of survival at 3 years and 5 years. **(C)** AUC analysis to evaluate the prognostic value of *CASP6* expression in glioma.

**Figure 3:**
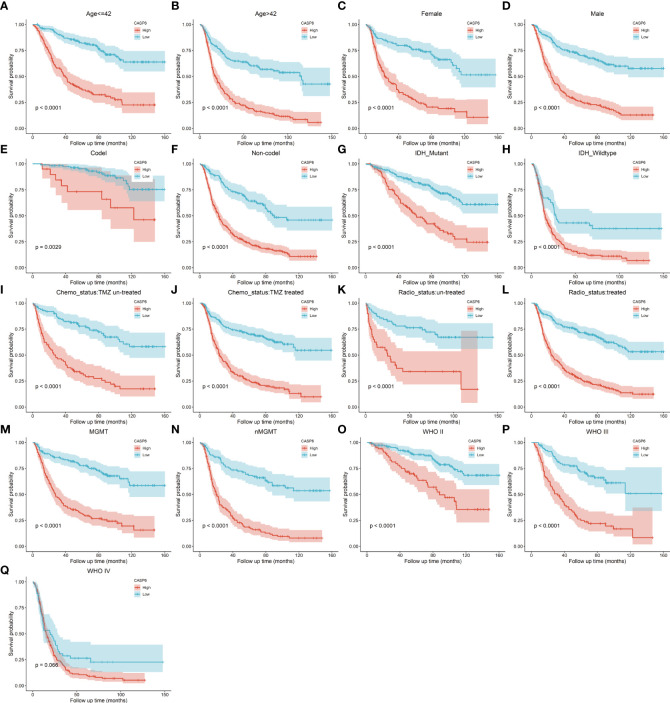
**(A–Q)** Stratified survival analysis of patients with low and high *CASP6* expression in the CGGA database, by age, sex, 1p19q codeletion, IDH mutation, chemotherapy status, radiotherapy status, MGMT status, and grade.

To explore the prognostic significance of *CASP6* in patients with glioma, we chose the CGGA database for further analyses (**Table 1**). Based on the median *CASP6* expression level, glioma samples were sub-classified into a group with high *CASP6* expression levels and a group exhibiting low levels of *CASP6* expression. Kaplan-Meier survival curve showed that patients with glioma with lower *CASP6* had a longer OS (*P* < 0.0001) (**Figure 2A**). The accuracy of *CASP6* expression to predict the 3-year-OS and 5-year-OS of patients with glioma was evaluated using a ROC curve. The AUC values for 3-, and 5-year OS were 0.733, and 0.759, respectively (**Figure 2B**). Consistently, analysis of TCGA and the REMBRANDT cohorts demonstrated that patients with glioma exhibiting lower *CASP6* expression levels had longer survival (*P* < 0.0001) (**Supplementary Figures 2A**, **3A**). In ROC curves based on the 3-, and 5-year OS, groups of patients with glioma exhibiting higher *CASP6* expression were separated from those with glioma of lower *CAP6* expression, with AUC values ranging from 0.668 to 0.809 (**Supplementary Figures 2B**, **3B**). Moreover, a 10-year time-dependent AUC was plotted to define the accuracy of different variables in predicting the OS of patients in the CGGA, TCGA, and REMBRANDT cohorts. Compared to AUC values based on gender, isocitrate dehydrogenase (IDH) activity, 1p19q codeletion, and *MGMT* (encoding O-6-methylguanine-DNA methyltransferase) gene promoter methylation, *CASP6* expression levels, age, and grade consistently showed higher AUC scores (**Figure 2C**; **Supplementary Figures 2C**, **3C**). In agreement, univariate and multivariate Cox analysis showed that *CASP6* expression could be a predictor of prognosis in patients with glioma (**Table 2**). Furthermore, the CGGA dataset was categorized according to age, gender, chemotherapy, radiotherapy, WHO grade, IDH mutation, 1p19q co-deletion, and *MGMT* methylation status. Each category was classified in into high-expression or low-expression groups based the median *CASP6* expression levels. Stratified survival analyses verified that low *CASP6* expression in each subgroup of patients was associated with longer survival (**Figure 3**). Accordingly, similar results were obtained from analysis of the TCGA and REMBRANDT databases (**Supplementary Figures 4**, **5**) Taken together, these data indicated that *CASP6* may represent a potential prognostic biomarker for patients with glioma.

**Table 1:** Clinical characteristics of 651 patients with primary glioma in the CGGA dataset according to *CASP6* expression.

CASP6 expression level		High	Low	P-value
Number		325	326	
CASP6_mRNA (median[IQR])		3.13 [2.79, 3.51]	1.81 [1.22, 2.12]	<0.001
Age (%)	≤42	133 (40.9)	181 (55.5)	<0.001
	>42	191 (58.8)	145 (44.5)	
Gender (%)	Female	130 (40.0)	136 (41.7)	0.714
	Male	195 (59.7)	190 (58.3)	
Grade (%)	II	70 (21.5)	162 (49.7)	<0.001
	III	87 (26.8)	107 (32.8)	
	IV	168 (51.7)	57 (17.5)	
Histology (%)	A (Astrocytoma)	57 (17.5)	74 (22.7)	<0.001
	AA (Anaplastic Astrocytoma)	73 (22.5)	47 (14.4)	
	AO (Anaplastic Oligodendroglioma)	14 (4.3)	44 (13.5)	
	AOA (Anaplastic Oligoastrocytoma)	0 (0.0)	16 (4.9)	
	GBM	168 (51.7)	57 (17.5)	
	O (Oligodendroglioma)	11 (3.4)	82 (25.2)	
	OA (Oligoastrocytoma)	2 (0.6)	6 (1.8)	
Survival (median[IQR])		22.7 [11.9, 51.8]	66.1 [30.2, 93.7]	<0.001
Status (%)	Alive	73 (22.5)	208 (63.8)	<0.001
	Dead	244 (75.1)	105 (32.2)	
IDH status (%)	Wildtype	215 (66.2)	72 (22.1)	<0.001
	Mutant	107 (32.9)	217 (66.6)	
1p19q (%)	Non-codel	302 (92.9)	144 (44.2)	<0.001
	Codel	22 (6.8)	120 (36.8)	
MGMTp methylation status (%)	un-methylated	148 (45.5)	114 (35.0)	0.037
	methylated	140 (43.1)	156 (47.9)	
Radio status (%)	No	43 (13.2)	71 (21.8)	0.006
	Yes	266 (81.8)	240 (73.6)	
Chemo status (%)	No	88 (27.1)	113 (34.7)	0.036
	Yes	217 (66.8)	191 (58.6)	

**Table 2:** Univariate and multivariate analysis of *CASP6* and clinical features in the CGGA datasets.

Univariate Cox analysis of CGGA (n=1018)			Multivariate Cox analysis CGGA (n=1018)		
Variables	*p* value	HR (95%CI)	Variables	*p* value	HR (95%CI)
CASP6	7.52E-30	2.1 (1.85-2.38)	CASP6	1.70E-06	1.64 (1.34-2.01)
Age	2.53E-21	1.04 (1.04-1.05)	Age	4.83E-05	1.02 (1.01-1.03)
Gender (Male vs. Female)	0.565	1.07 (0.86-1.32)	Gender (Male vs. Female)	0.861	0.98 (0.76-1.26)
Grade (WHOIV vs. WHOIII vs. WHOII)	2.65E-48	2.99 (2.58-3.46)	Grade (WHOIV vs. WHOIII vs. WHOII)	4.83E-09	1.89 (1.53-2.34)
Radio-status (Treated vs. Untreated)	0.0429	1.38 (1.01-1.88)	Radio-status (Treated vs. Untreated)	0.447	0.87 (0.6-1.26)
Chemo-status (Treated vs. Untreated)	0.0286	1.3 (1.03-1.65)	Chemo-status (Treated vs. Untreated)	0.00106	0.61 (0.46-0.82)
IDH (Mutant vs. Wildtype)	1.78E-37	0.22 (0.18-0.28)	IDH (Mutant vs. Wildtype)	0.127	0.77 (0.54-1.08)
1p19q (Codeletion vs. non-Codeletionl)	1.44E-19	0.14 (0.09-0.21)	1p19q (Codeletion vs. non-Codeletionl)	7.25E-05	0.35 (0.21-0.59)
MGMT (methyltransferase vs. non- methyltransferase	0.000936	0.68 (0.55-0.86)	MGMT (methyltransferase vs. non- methyltransferase	0.0353	0.76 (0.59-0.98)

HR, hazard ratio; IDH, isocitrate dehydrogenases; MGMT, O-6-methylguanine-DNA methyltransferase.

### *CASP6* expression could predict differences in TIME

**Figure 4:**
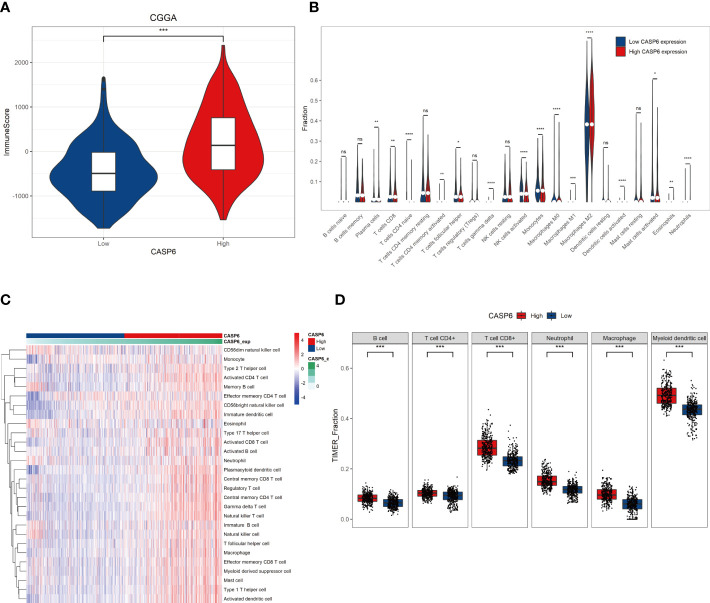
Relationships with *CASP6* in the tumor immune microenvironment from the CGGA database. **(A)** Correlation analysis of the ImmuneScore and *CASP6* levels. **(B)** The levels of infiltration of 22 types of immune cells in the low and high *CASP6* expression groups. **(C)** Results of ssGSEA analysis of CASP6 in the CGGA database. **(D)** Results of TIMER analysis of CASP6 in the CGGA database. ^*^ *P* < 0.05, ^**^ *P* < 0.01, ^***^ *P* < 0.001, ^****^ *P* < 0.0001. ns, no significance.

**Figure 5:**
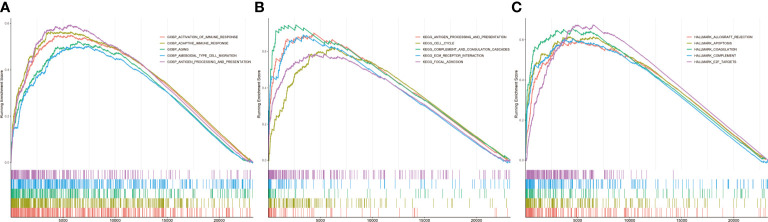
Related pathways analyzed by GSEA in the CGGA database. **(A)** GO analysis of *CASP6*. **(B)** KEGG analysis of *CASP6*. **(C)** Hallmark analysis of *CASP6*.

Infiltration and activation of immune cells is associated with the prognosis of glioma (23). To investigate the role of CASP6 in the TIME of glioma, we evaluated the immune score and immune infiltration in glioma samples with low or high *CASP6* expression levels, respectively. In both CGGA and TCGA datasets, glioma sample group with increased levels of *CASP6* expression exhibited a higher immune score than the group with decreased *CASP6* expression (**Figure 4A**; **Supplementary Figure 6A**). The presence of immune cells and their identity in the CGGA and TAGA cohorts were analyzed using the CIBERSORT, ssGSEA, and TIMER algorithms. Compared to glioma sample group with lower *CASP6* expression levels, the proportions of naïve T cells, activated natural killer (NK) cells, and M0 macrophages were markedly decreased in glioma samples exhibiting higher levels of *CASP6* expression, whereas the proportions of gamma delta T cells, monocytes, M2 macrophages, activated dendritic cells, and neutrophils were significantly increased in this group (**Figures 4B–D**; **Supplementary Figures 6B–D**). To understand the effects of *CASP6* expression on TIME, we investigated the biological functions of CASP6. GO analysis showed that CASP6 was mainly involved in processes including “activation of immune response”, “adaptive immune response”, “aging”, “ameboidal cell migration”, and “antigen processing and presentation” (**Figure 5A**). Furthermore, the annotations of the KEGG pathway revealed an enrichment of CASP6 in pathways including “antigen processing and presentation”, “cell cycle”, “complement and coagulation cascades”, “receptor interaction”, and “focal adhesion” (**Figure 5B**). GSEA analysis showed that higher expression of *CASP6* was associated with hallmarks of tumorigenesis including “apoptosis”, “allograft rejection”, “coagulation”, “complement”, and “E2F targets” correlated markedly (**Figure 5C**; **Supplementary Table 3**). Taken together, these results indicated that CASP6 may play a role in regulating in immune cell infiltration in glioma.

### CASP6 could serve as a biomarker to predict response to temozolomide and immunotherapy

**Figure 6:**
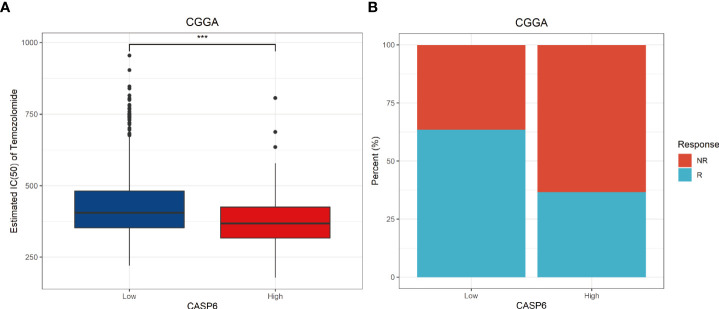
Potential predictive value of *CASP6* in chemotherapy and immunotherapy in the CGGA database. **(A)** IC50 of temozolomide in the low and high *CASP6* expression groups. Low *CASP6* expression group (n = 326), high *CASP6* expression group (n = 325) **(B)** Immunotherapy responses in the low and high *CASP6* expression groups. Low *CASP6* expression group [response: n = 207, (63.50%); non-response: n = 119, (36.50%)], high *CASP6* expression group (response: n = 119, (36.62%); non-response: n = 206, (63.38%)) ^***^ *P* < 0.001.

As revealed by the GO and GSEA analyses, CASP6 was associated with “activation of the immune response”, “cell cycle”, and “apoptosis” processes. Thus, we investigated the predictive value of CASP6 expression in the response to temozolomide and immunotherapy. Temozolomide (TMZ) is one of the most common chemotherapeutic options for glioma treatment (24), and has been shown to improve the survival rate of patients newly diagnosed with glioma (25). Nonetheless, resistance to TMZ remains a conundrum in glioma chemotherapy. Simultaneously, immunotherapy has been increasingly applied to patients with glioma in recent years. Finding suitable molecular characteristics to predict the efficacy of immunotherapy is urgently required. We calculated the IC50 of TMZ associated with *CAPS6* expression to estimate its role in selecting the best treatment methods. Notably, TMZ presented a better therapeutic response in patients with glioma with high *CASP6* expression (**Figure 6A**). The outcome of TMZ response prediction in the TCGA cohort was consistent with that of the CGGA cohort (**Supplementary Figure 8A**). Meanwhile, the TIDE results further predicted that patients with high expression of *CASP6* would achieve a poorer response to immunotherapy than those with low *CASP6* expression (**Figure 6B**). Our findings revealed that the CASP6 could play a role in determining therapeutic strategies for patients with glioma.

### Meta-analysis of *CASP6* and validation of *CASP6* expression in patients with glioma and cells

**Figure 7:**
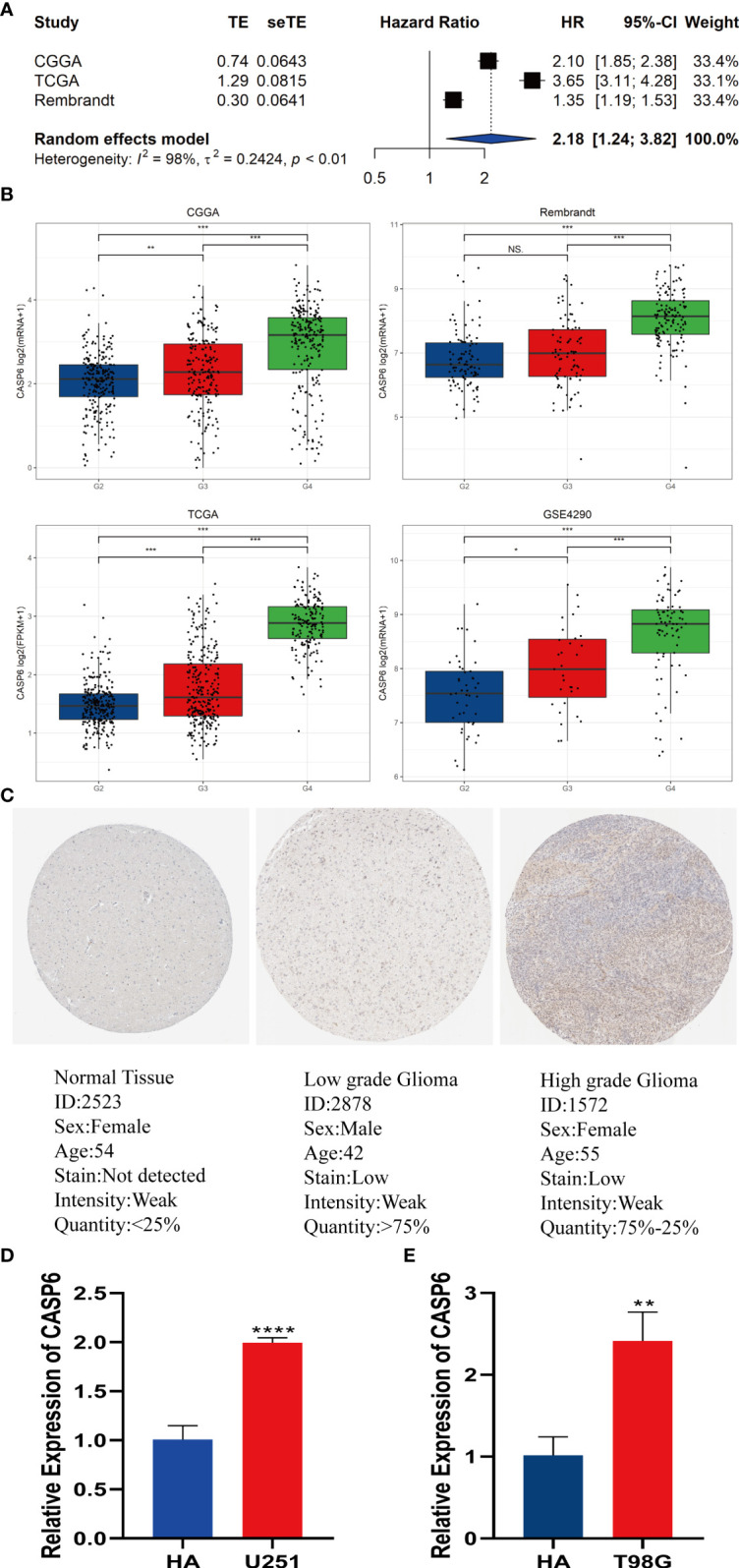
Meta-analysis of *CASP6* and its expression level in gliomas. **(A)** Meta-analysis of *CASP6* in the CGGA, TCGA and REMBRANDT databases. **(B)** *CASP6* expression increased with disease progression of glioma (G2: Grade2, G3: Grade3, G4:Grade4). **(C)** Differentially expression of CASP6 in glioma and normal tissues in The Human Protein Atlas database. **(D)** qRT-PCR indicated that the expression of *CASP6* was upregulated in U251 cells compared with that in HAs. **(E)** qRT-PCR indicated that the expression of CASP6 was upregulated in T98G cells compared with that in HA. ^*^ *P* < 0.05, ^**^ *P* < 0.01, ^***^ *P* < 0.001, ^****^ *P* < 0.0001. NS: no significance.

To improve the reliability of the results, a meta-analysis of the CGGA, TCGA, and REMBRANDT datasets was performed. The results confirmed that patients with high expression of *CASP6* had a shorter OS than patients with lower *CASP6* expression (HR = 2.18, 95% CI 1.24–3.82, **Figure 7A**). Further analysis of *CASP6* expression datasets obtained from the CGGA, REMBRANDT, TCGA, GSE4290 databases showed that higher *CASP6* expression was associated with the grade of glioma (**Figure 7B**). Immunohistochemical images of normal brain tissue, low-grade glioma, and high-grade glioma acquired from the HPA, confirmed that the protein level of CASP6 increased with increasing tumor grade (**Figure 7C**). Finally, qRT-PCR analysis revealed that the *CASP6* mRNA content in glioma cells (U251 and T98G) was almost two-fold higher than that in normal astrocyte cells (HA) (**Figures 7D, E**)

### Knocking down of *CASP6* inhibits the proliferation of glioma cells

**Figure 8:**
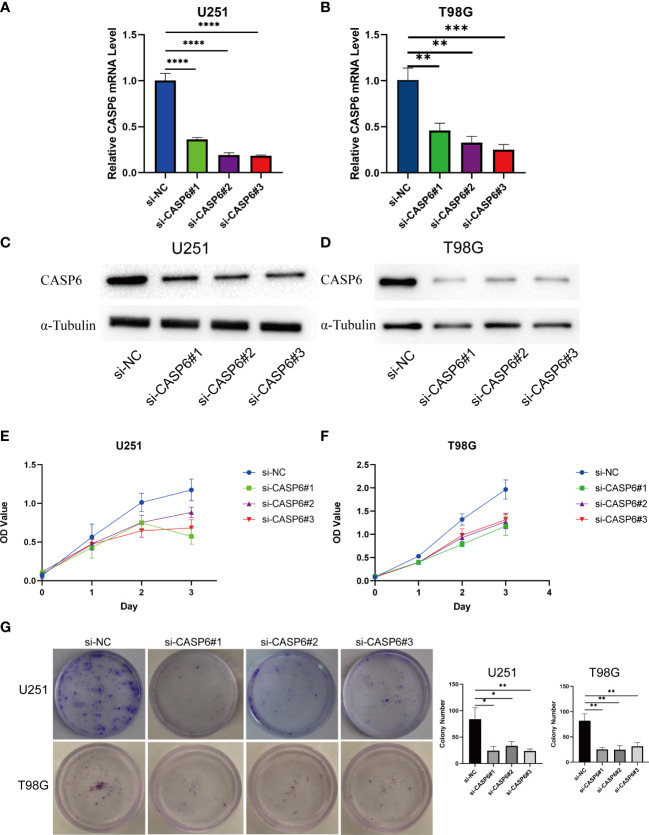
Results of loss of function experiments in U251 and T98G glioma cell lines. **(A, B)** The *CASP6* knockdown efficiency of different siRNAs in U251 and T98G cells. **(C, D)** Identification of *CASP6* knockdown efficiency by western blotting. **(E, F)** CCK-8 assays of U251 and T98G glioma cell lines knocked down for *CASP6*. **(G)** Colony formation assays of U251 and T98G glioma cell lines knocked down for *CASP6*. *P < 0.05, **P < 0.01, ***P < 0.001, ****P < 0.0001.

CCK-8 and colony formation assays were conducted to evaluate the effects of knocking down *CASP6* expression on glioma cell proliferation. The efficiency of *CASP6* knockdown was confirmed using qRT-PCR and western blotting. All three siRNAs significantly reduced the expression of CASP6 in U251 and T98G cell lines (**Figures 8A–D**). The CCK-8 assay showed that *CASP6* knockdown dramatically inhibited the proliferation of U251 and T98G cells (**Figures 8E, F**). Colony forming assays showed that the colony-forming capacity of U251 and T98G cells was reduced significantly after *CASP6* knockdown (**Figure 8G**). Collectively, these results demonstrated that the expression of *CASP6* correlated positively with glioma cell proliferation.

## Discussion

Glioma is the most common primary brain neoplasm and is a leading cause of cancer-associated death worldwide (26). Recently, the therapeutic approaches to glioma have improved significantly, however, the clinical outcomes of patients with glioma remain poor (27). Immunotherapy can be effective in many tumors; however, the factors that influence the efficacy of immunotherapy remain complex and relatively unknown (28).

Pyroptosis plays a pivotal role in the onset and development of various diseases (29–31). Interestingly, pyroptosis plays conflicting roles in the promotion and inhibition of oncogenesis and the tumor microenvironment (32, 33). Furthermore, PRG-related prognostic models have been constructed for many neoplasms, including gastric cancer (34), skin cutaneous melanoma (35), breast cancer (36), and thyroid cancer (37). Previously, a prognostic model comprising three PRGs (*CASP4*, *CASP9*, and *NOD2* (encoding nucleotide binding oligomerization domain containing 2)) was constructed to predict the outcomes of patients with glioma (38). The results of the present study are more comprehensive because of the precise construction of the prognosis model, which was based on more databases, bioinformatic analysis and *in vitro* experiments.

CASP6 is an apoptotic caspase (39) that is involved in multiple cell death pathways. It can promote the activation of programmed cell death pathways including pyroptosis, apoptosis, and necroptosis (PANoptosis) (40). However, the status of *CASP6* as a PRG in glioma has been rarely reported (12, 41), therefore, the role of CASP6 in glioma was unclear. Our results showed that *CASP6* was a significant biomarker to predict the prognosis of patients with glioma. In the OS analysis, patients with lower *CASP6* expression experienced longer survival. ROC analysis demonstrated that *CASP6* expression was a reliable marker to predict clinical outcomes in patients with glioma. Indeed, stratification survival analysis demonstrated the good predictive role of *CASP6* expression in glioma. Furthermore, univariate and multivariate Cox analyses identified *CASP6* expression as an independent prognostic risk factor for glioma patients.

PRGs regulate the TIME through various mechanisms (42, 43). According to the TME prognostic models, which are based on the characteristics of 33 cancers in the TCGA database, six “Immune Subtype” clusters (C1-C6) were identified (44). Glioma was grouped in cluster C4 (Lymphocyte Depleted), with features of a repressed Th1 response and a high M2 response. Conversely, LGG was classified into cluster C5 (Immunologically Quiet). Cluster C5 cluster has the lowest lymphocyte infiltration and the highest M2 macrophage responses. To explore the effects of CASP6 on the brain immune microenvironment of patients with glioma, we used several deconvolution algorithms. The ESTIMATE findings indicated that the high *CASP6* expression group had a higher ImmuneScore, which meant that immune cell infiltration was higher in the CASP6 high-expression group in the TIME. The proportion of M2 macrophages increased markedly in the high *CASP6* expression group. These results are consistent with the features of immune subtype clusters C4 and C5, and may support a malignant biological behavior of glioma cells (45). Conversely, patients with glioma with a higher proportion of regulatory T cell (Treg) infiltration were consistently associated with poor prognosis (46). Our findings support this conclusion. The high *CASP6* expression group, which was associated with poorer prognosis, exhibited higher infiltration of Tregs.

Considering the differences in the TIME between the two groups, we conducted GO and KEGG functional enrichment analyses to identify the underlying regulatory mechanism, which implied that CASP6 was primarily associated with the immune response and focal adhesion.

TMZ, the most common chemotherapeutic agent used to treat gliomas, can significantly prolong the survival of patients with glioma. However, the response to chemotherapy varies across individuals. To estimate the predictive value of *CASP6* expression in clinical therapy, sensitivity to TMZ was calculated based on gene expression profiles. The results indicated that patients with high *CASP6* expression were more sensitive to TMZ.

Studies have validated the importance of immune cell infiltration in patients with glioma (46). Immunotherapies can markedly improve patient survival and have shown significant antitumor outcomes in several clinical trials (47). The TME of glioma is shaped by the disease itself and not by the surrounding brain tissue. The innate immune system, instead of CD8+T cells, might have greater responsibility for the therapeutic effects of anti- programmed cell death-1 (PD-1) antibodies in glioblastoma. In glioblastoma, severe T cell exhaustion induced upregulation of multiple immune checkpoints, which inhibits immune modulation (48). Furthermore, not all patients with glioma can benefit from monotherapy immune checkpoint inhibition (49). Therefore, new predictive biomarkers to improve precision immunotherapy for patients with glioma are required. In our study, patients with glioma with lower *CASP6* expression presented a better response to immunotherapy.

To further confirm the predictive value of *CASP6* expression as a new prognostic biomarker for glioma, we conducted a meta-analysis, which showed that based on its HR and 95% CI (2.18 and 1.24–3.82, respectively), *CASP6* expression is a robust prognostic indicator.

In addition, we performed experimental validation of aberrant *CASP6* expression in patients with different grades of glioma. The bioinformatics analysis showed that *CASP6* expression increased with the increasing degree of malignancy of glioma. The immunohistochemical images obtained from the HPA showed that CASP6 expression was lower in normal brain tissue than in glioma tissue. *CASP6* expression in normal human astrocytes was lower than that in human glioma cell lines (U251 and T98G), as confirmed by qRT-PCR. Furthermore, the HPA immunohistochemistry images showed that high-grade glioma tissues contained higher levels of CASP6 than low-grade glioma tissue. Thus, the above observations confirmed the findings of the bioinformatics analysis.

Finally, *in vitro* functional experiments showed that knockdown of *CASP6* inhibited the proliferation of glioma cells. CCK-8 and colony forming assays demonstrated that CASP6 is highly related to the proliferation of glioma. Thus, CASP6 might represent a potential target in the treatment of glioma.

In this study, we selected the CGGA as the validation cohort, because it is the largest Chinese sample database, containing clinical and follow-up information of patients with glioma. Furthermore, we excluded patients with secondary and recurrent glioma because of their complex biological characteristics. Nonetheless, the immunotherapy response of patients showed opposite trends when comparing the TCGA data with the CGGA data (**Supplementary Figure 8B**). We suspect that differences in ethnicities might be responsible for these contrasting results.

## Conclusion

In the present study, we identified the PRDEG CASP6 as a biomarker for glioma. We propose that detecting *CASP6* expression combined with clinical features might improve the diagnostic accuracy in patients with glioma. *In vitro*, *CASP6* was verified as an oncogene in glioma, and CASP6 inhibition prevented glioma cell proliferation. The results of the present study might promote innovative strategies to assess immunotherapy outcomes and thus improve the prognosis of patients with glioma.

## Data availability statement

The original contributions presented in the study are included in the article/**Supplementary Material**. Further inquiries can be directed to the corresponding author.

## Author contributions

ZZ oversaw the overall design of this research. KG performed the experiments, analyzed the data, and wrote the manuscript. JZ contributed to the R software analysis and provided R language modification. QJ verified the data analysis. HY revised the figures and tables. YS revised the discussion of the article. All authors contributed to the article and approved the submitted version.

## Funding

This work was supported by the National Key R & D Program Intergovernmental Cooperation on International Scientific and Technological Innovation of the Ministry of Science and Technology of China [grant number 2017YFE0110400]; the National Natural Science Foundation of China [grant number 81870984]; the Special Project for the Construction of Hebei Province International Science and Technology Cooperation Base [grant number 193977143D]; the Government Funded Project on Training of Outstanding Clinical Medical Personnel and Basic Research Projects of Hebei Province in the Year of 2019; and the Medical science research project of Hebei Province [grant number 20201567].

## Acknowledgments

The authors would like to thank the CGGA, REMBRANDT, TCGA, GTEx and GEO databases for providing the data.

## Conflict of interest

The authors declare that the research was conducted in the absence of any commercial or financial relationships that could be construed as a potential conflict of interest.

## Publisher’s note

All claims expressed in this article are solely those of the authors and do not necessarily represent those of their affiliated organizations, or those of the publisher, the editors and the reviewers. Any product that may be evaluated in this article, or claim that may be made by its manufacturer, is not guaranteed or endorsed by the publisher.

## Abbreviations

AUC, Area under the curve; CCK-8, Cell Counting Kit-8; CNS, Central nervous system; CGGA, Chinese Glioma Genome Atlas; DEGs, Differentially expressed genes; ESTIMATE, Estimation of Stromal and Immune cells in Malignant Tumors using Expression data; GEO, Gene Expression Omnibus; GSE, Gene Expression Omnibus Series; GO, Gene Ontology; GSEA, Gene Set Enrichment Analysis; GDSC2, Genomics of Drug Sensitivity in Cancer 2; GTEx, Genotype-Tissue Expression; GBM, Glioblastoma; IC50, Half maximal inhibitory concentration; HR, Hazard ratio; HA, Human astrocytes; HPA, Human Protein Atlas; ICB, Immune checkpoint blockade; KEGG, Kyoto Encyclopedia of Genes and Genomes; OS, Overall survival; PRDEGs, Pyroptosis-related differential expressed genes; PRGs, Pyroptosis-related genes; qRT-PCR, Quantitative real-time reverse transcription PCR; ROC, Receiver operating characteristic; Tregs, Regulatory T cells; REMBRANDT, Repository for Molecular Brain Neoplasia Data; ssGSEA, Single sample gene set enrichment analysis; TMZ, Temozolomide; TCGA, The Cancer Genome Atlas; TIDE, Tumor Immune Dysfunction and Exclusion; TIME, Tumor immune microenvironment; TIMER, Tumor Immune Estimation Resource; TME, Tumor microenvironment; WHO, World Health Organization.
