# Supplementary figures and images for: CASP6 predicts poor prognosis in glioma and correlates with tumor immune microenvironment
Source: Front Oncol. 2022 Sep 2;12:818283. doi: 10.3389/fonc.2022.818283 (PMC9479196; doi:10.3389/fonc.2022.818283)

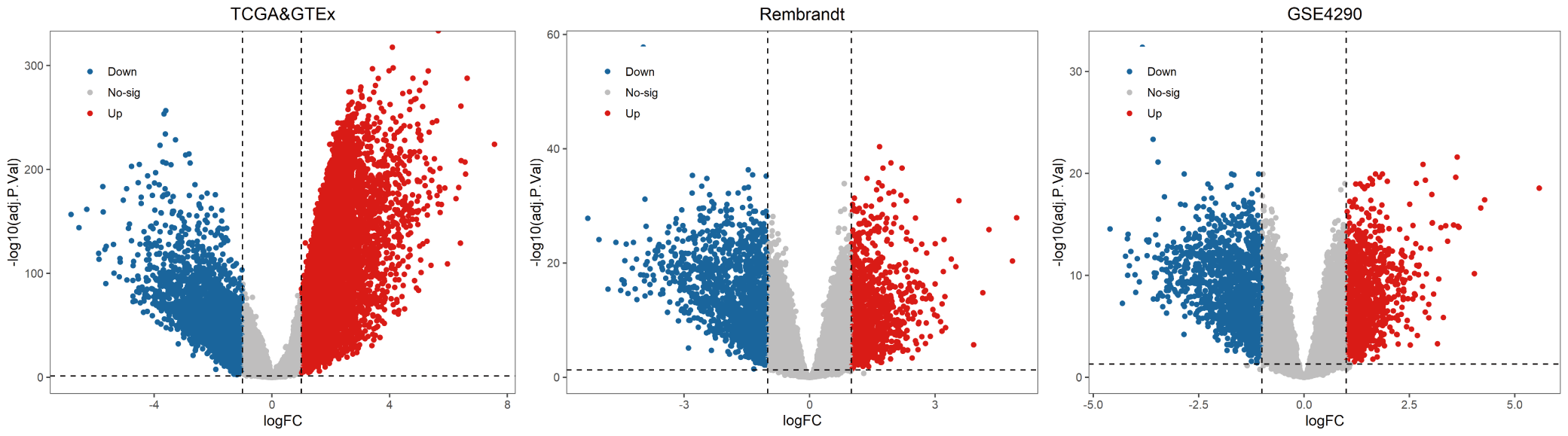

Supplement: Supplementary file 1 [file Image_1.tif]

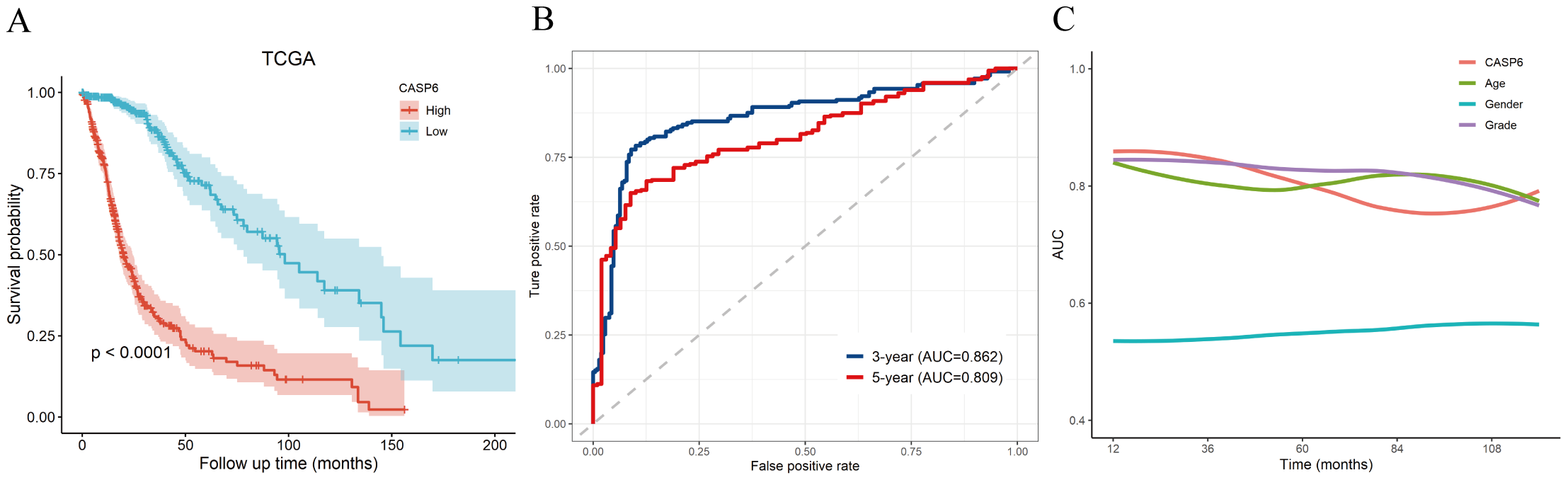

Supplement: Supplementary file 2 [file Image_2.tif]

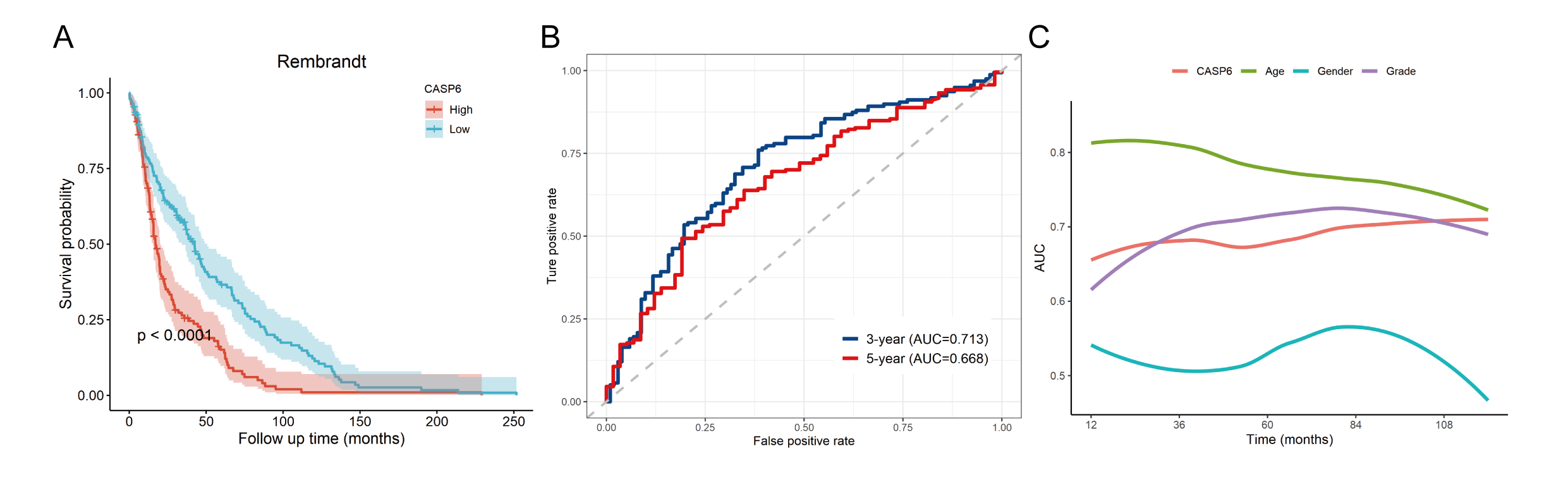

Supplement: Supplementary file 3 [file Image_3.tif]

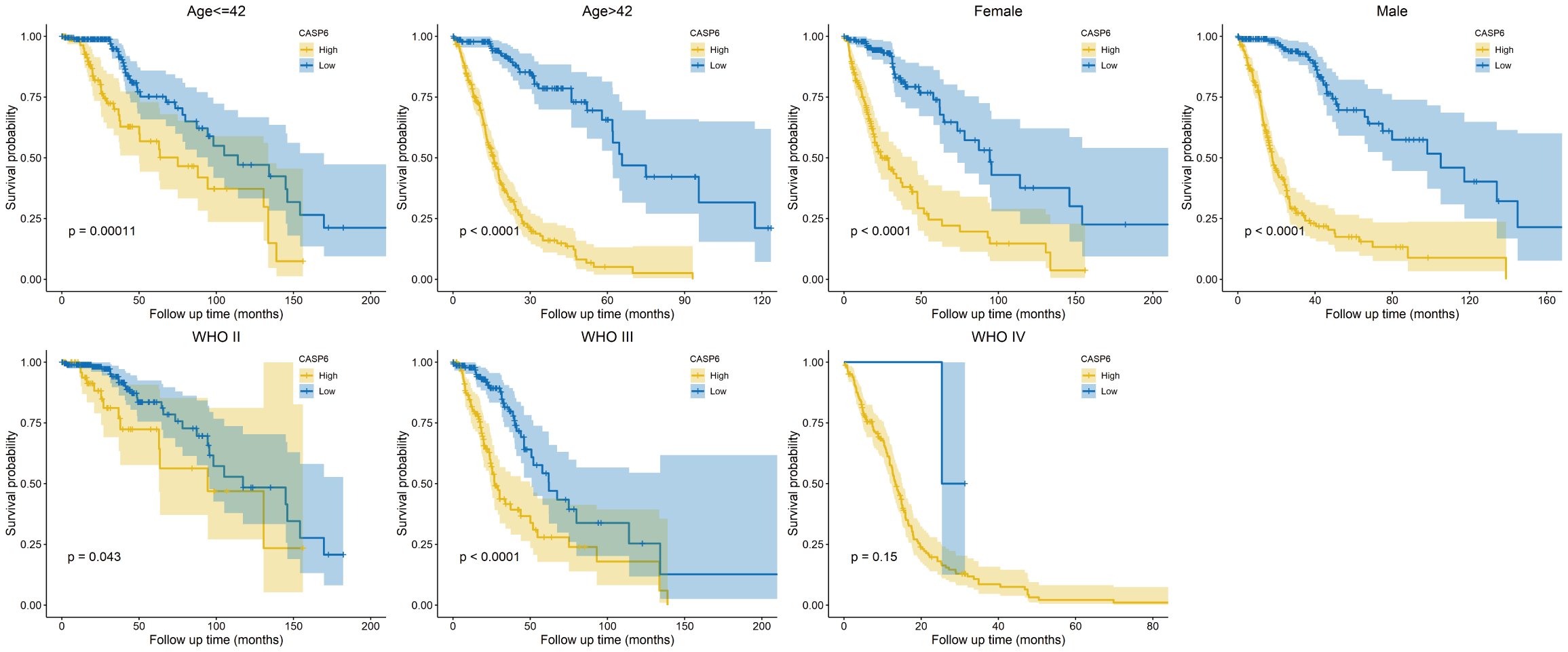

Supplement: Supplementary file 4 [file Image_4.tif]

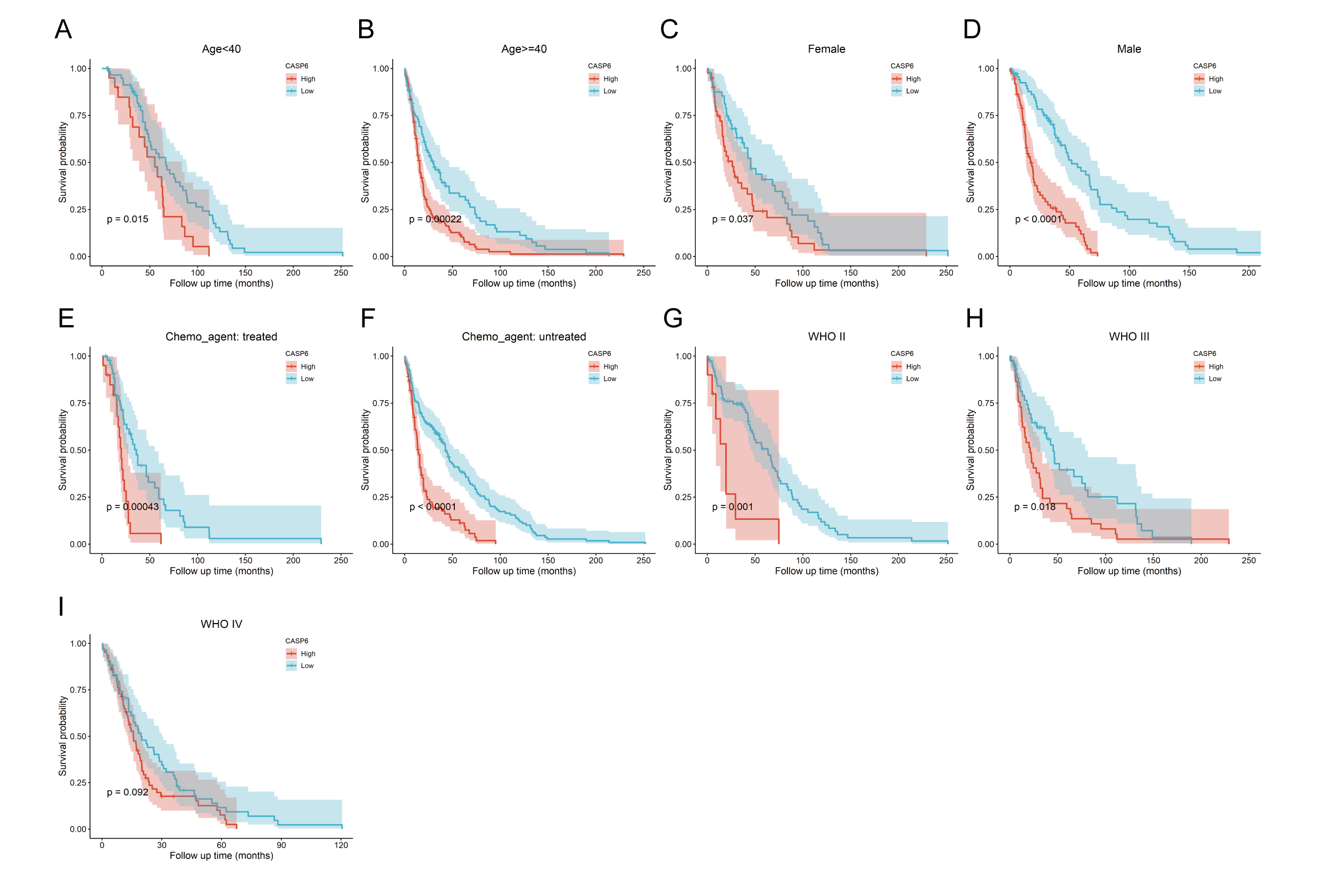

Supplement: Supplementary file 5 [file Image_5.tif]

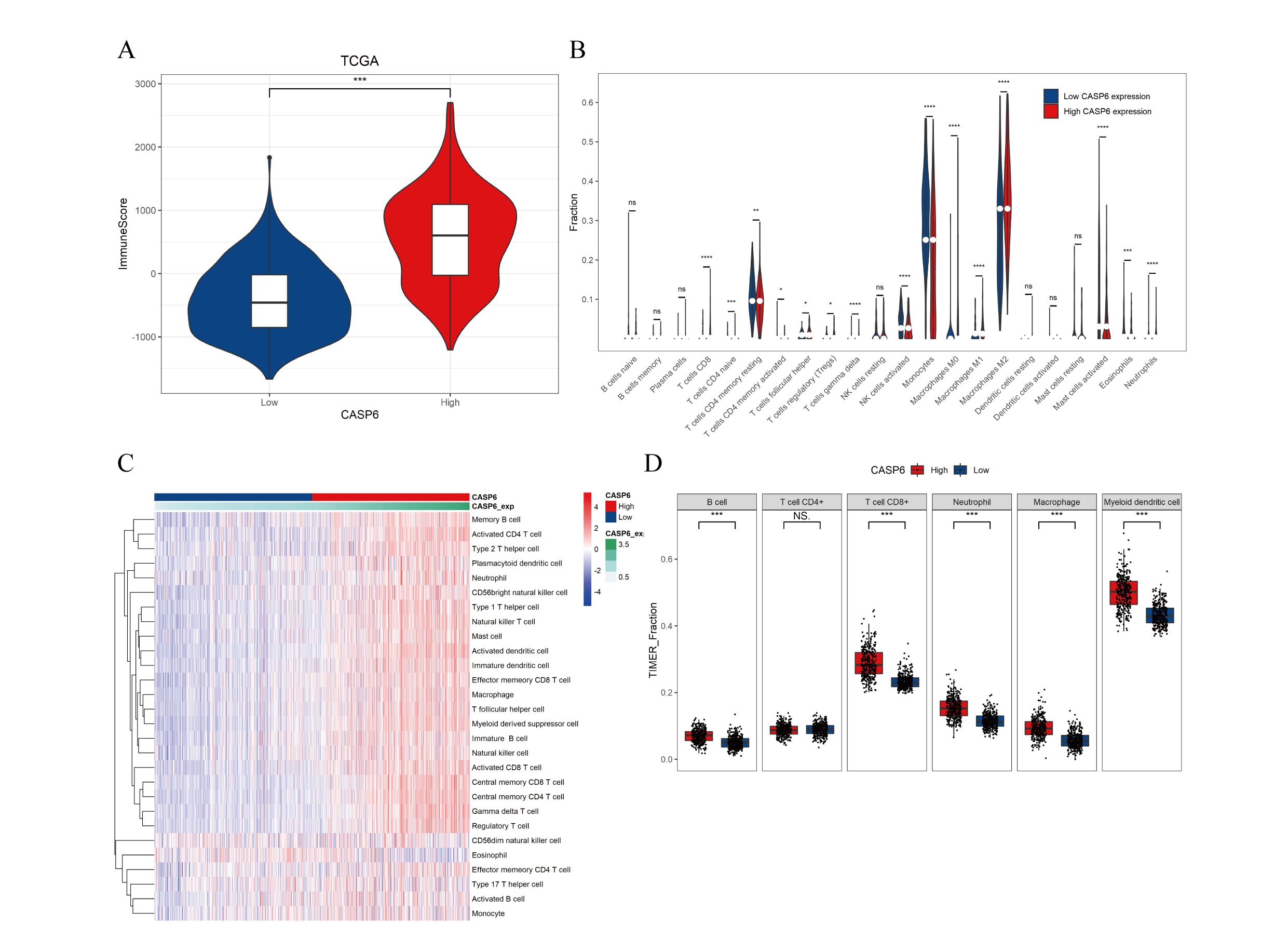

Supplement: Supplementary file 6 [file Image_6.tif]

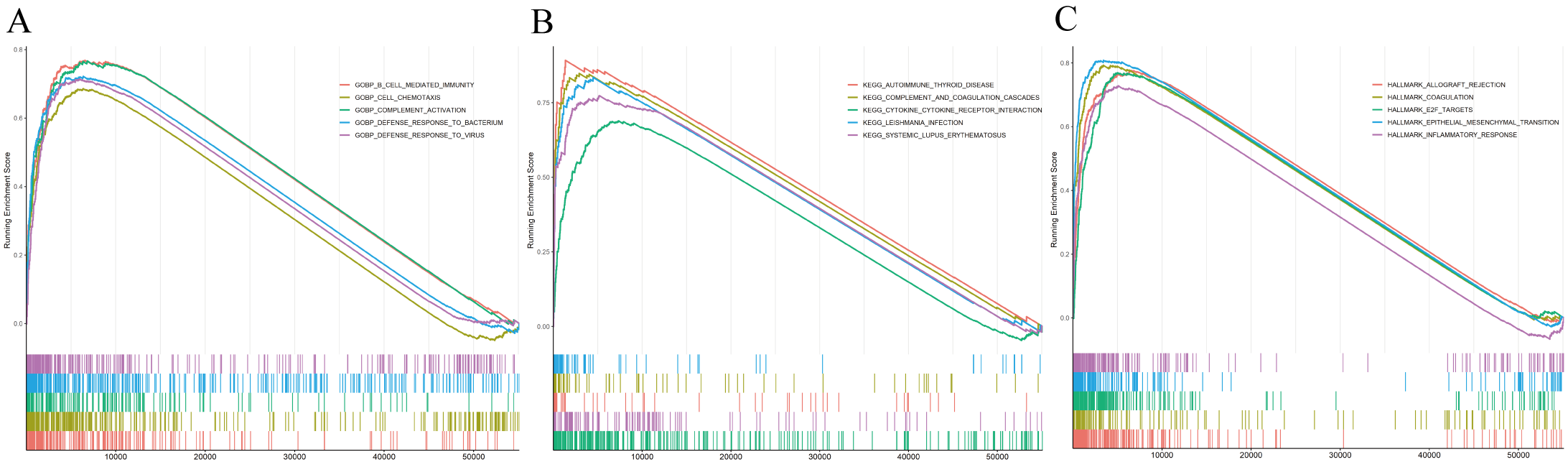

Supplement: Supplementary file 7 [file Image_7.tif]

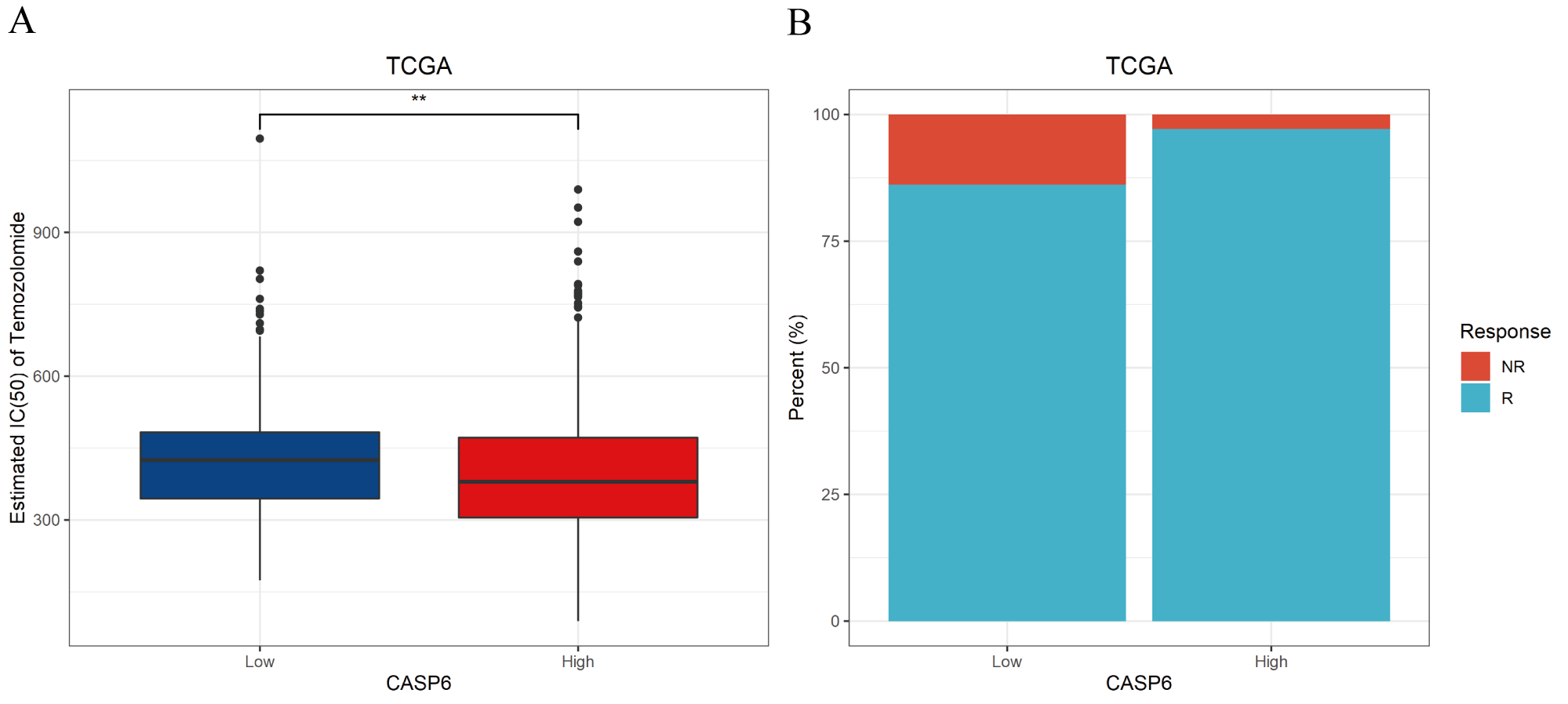

Supplement: Supplementary file 8 [file Image_8.tif]

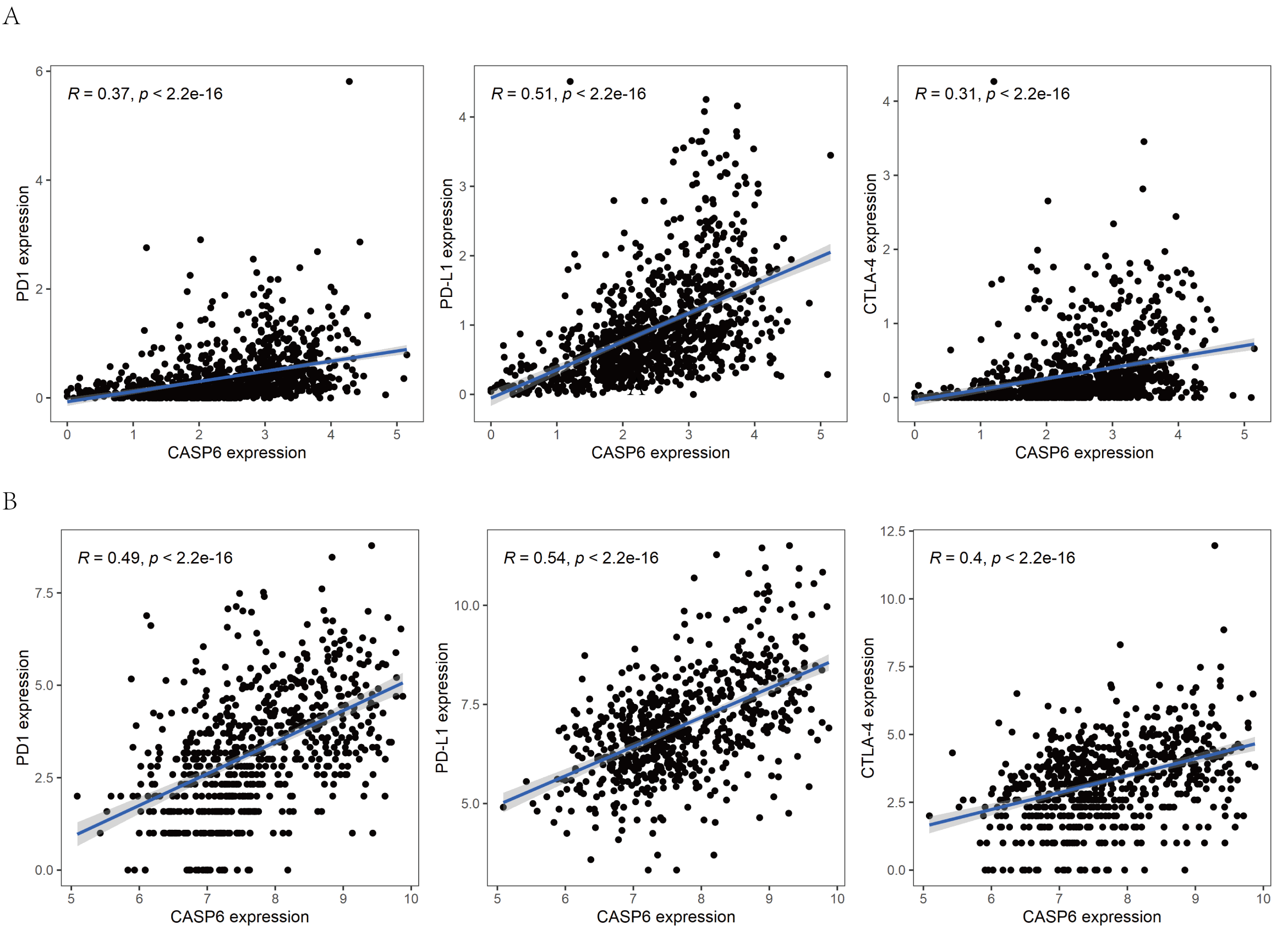

Supplement: Supplementary file 9 [file Image_9.tif]

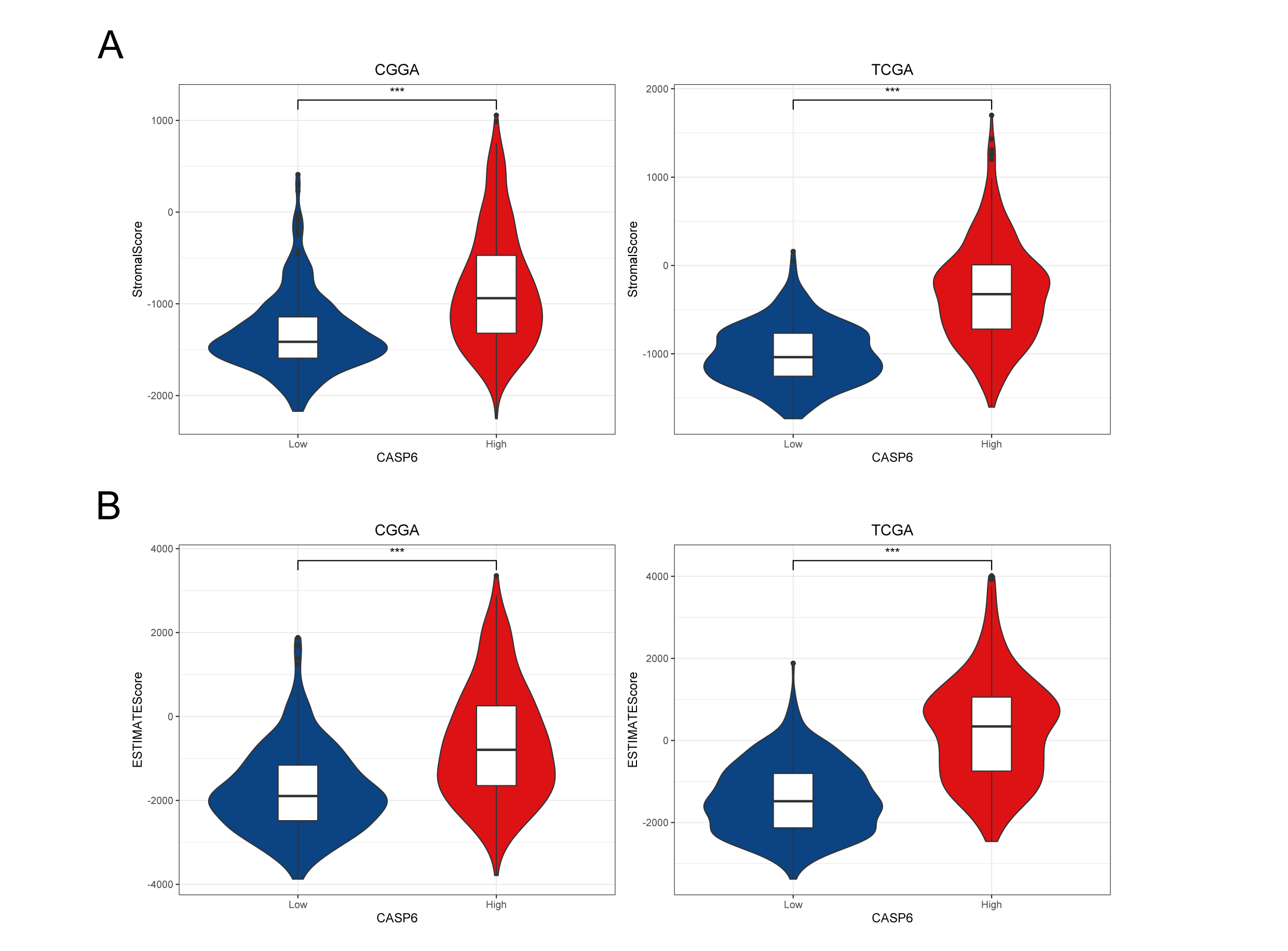

Supplement: Supplementary file 10 [file Image_10.tif]
