# Supplementary material for: CASP6 predicts poor prognosis in glioma and correlates with tumor immune microenvironment
Source: Front Oncol. 2022 Sep 2;12:818283. doi: 10.3389/fonc.2022.818283 (PMC9479196; doi:10.3389/fonc.2022.818283)
Supplement: Supplementary file 11 [file Table_1.docx]

**Supplementary Table1:** Pyroptosis-Related Genes

| GSDMD | PTEN | NAIP | GBP1 | TET2 | BSG | GZMA |
| --- | --- | --- | --- | --- | --- | --- |
| GSDME | ADORA1 | DHX9 | GJA1 | UTS2 | IL32 | DPP9 |
| NLRP3 | ADORA2B | NLRP9 | PRDM1 | CTSV | MALT1 | DPP8 |
| CASP1 | ADORA2A | CASP3 | UBR2 | NFKB1 | STK4 | NLRC4 |
| GSDMB | ADORA3 | IL18 | CPTP | APOE | MST1 | CASP8 |
| CASP4 | PECAM1 | HMGB1 | TP53 | SDHB | PRF1 | CASP5 |
| GSDMC | METTL3 | APIP | VDR | EEF2K | ELAVL1 | AIM2 |
| NLRP1 | TRIM31 | TREM2 | BRD4 | P2RX7 | HDAC6 | ZBP1 |
| GSDMA | CAMP | FOXO3 | NEK7 | CD274 | SQSTM1 | PYCARD |
| CARD8 | MRE11 | CASP6 | CRTAC1 | FGF21 | IRF3 | STING1 |
| GZMB | PARP1 | TXNIP | NFE2L2 | CEBPB | ZDHHC1 | TFAM |
| IL1B | GBP5 | DDX3X | AGER |  |  |  |
