# Supplementary material for: CASP6 predicts poor prognosis in glioma and correlates with tumor immune microenvironment
Source: Front Oncol. 2022 Sep 2;12:818283. doi: 10.3389/fonc.2022.818283 (PMC9479196; doi:10.3389/fonc.2022.818283)
Supplement: Supplementary file 12 [file Table_2.docx]

**Supplementary Table 2:** Direct links to the immunohistochemistry images from the Human Protein Atlas

| Sample | Web Link |
| --- | --- |
| Normal | https://www.proteinatlas.org/ENSG00000138794-CASP6/tissue/cerebral+cortex#img |
| Glioma, low grade | https://www.proteinatlas.org/ENSG00000138794-CASP6/pathology/glioma#img |
| Glioma, high grade | https://www.proteinatlas.org/ENSG00000138794-CASP6/pathology/glioma#img |
